# Targeting DNA Damage Response Pathway in Ovarian Clear Cell Carcinoma

**DOI:** 10.3389/fonc.2021.666815

**Published:** 2021-10-19

**Authors:** Oscar G. W. Wong, Jing Li, Annie N. Y. Cheung

**Affiliations:** Department of Pathology, The University of Hong Kong, Hong Kong, China

**Keywords:** ovarian cancer (OC), ovarian clear cell carcinoma, DNA damage response (DDR), targeted therapy, ARID1A

## Abstract

Ovarian clear cell carcinoma (OCCC) is one of the major types of ovarian cancer and is of higher relative prevalence in Asians. It also shows higher possibility of resistance to cisplatin-based chemotherapy leading to poor prognosis. This may be attributed to the relative lack of mutations and aberrations in homologous recombination-associated genes, which are crucial in DNA damage response (DDR), such as *BRCA1*, *BRCA2*, *p53*, *RAD51*, and genes in the Fanconi anemia pathway. On the other hand, OCCC is characterized by a number of genetic defects rendering it vulnerable to DDR-targeting therapy, which is emerging as a potent treatment strategy for various cancer types. Mutations of *ARID1A*, *PIK3CA*, *PTEN*, and catenin beta 1 (*CTNNB1*), as well as overexpression of transcription factor hepatocyte nuclear factor-1β (*HNF-1β*), and microsatellite instability are common in OCCC. Of particular note is the loss-of-function mutations in *ARID1A*, which is found in approximately 50% of OCCC. *ARID1A* is crucial for processing of DNA double-strand break (DSB) and for sustaining DNA damage signaling, rendering *ARID1A*-deficient cells prone to impaired DNA damage checkpoint regulation and hence sensitive to poly ADP ribose polymerase (PARP) inhibitors. However, while preclinical studies have demonstrated the possibility to exploit DDR deficiency in OCCC for therapeutic purpose, progress in clinical application is lagging. In this review, we will recapitulate the preclinical studies supporting the potential of DDR targeting in OCCC treatment, with emphasis on the role of *ARID1A* in DDR. Companion diagnostic tests (CDx) for predicting susceptibility to PARP inhibitors are rapidly being developed for solid tumors including ovarian cancers and may readily be applicable on OCCC. The potential of various available DDR-targeting drugs for treating OCCC by drawing analogies with other solid tumors sharing similar genetic characteristics with OCCC will also be discussed.

## Introduction

Ovarian carcinoma is the most lethal gynecologic malignancy in many countries, with an estimated incidence of 290,000 cases and a mortality of 180,000 cases per year worldwide (1). Histologically, ovarian carcinomas are classified into several different subtypes, including the four major types: serous, endometrioid, clear cell, and mucinous (2). Each subtype exhibits distinct genetic alterations and clinical and prognostic characteristics. Ovarian clear cell carcinoma (OCCC) accounts for approximately 5~27% of all ovarian carcinomas (3–5). It is characterized by high recurrence rate and generally displays worse prognosis owing to frequent *de novo* resistance to chemotherapeutic agents in advanced stages (6, 7). A number of targeted therapy strategies, including anti-angiogenesis, multitargeted tyrosine kinase inhibition, and PIK3CA/AKT/mTOR pathway inhibition as well as immunotherapy were evaluated in clinical trials, but only modest efficacies or non-histotype selective effects were observed (8). Thus, discovering effective targeted therapies for OCCC remains a challenging goal.

**Figure 1:**
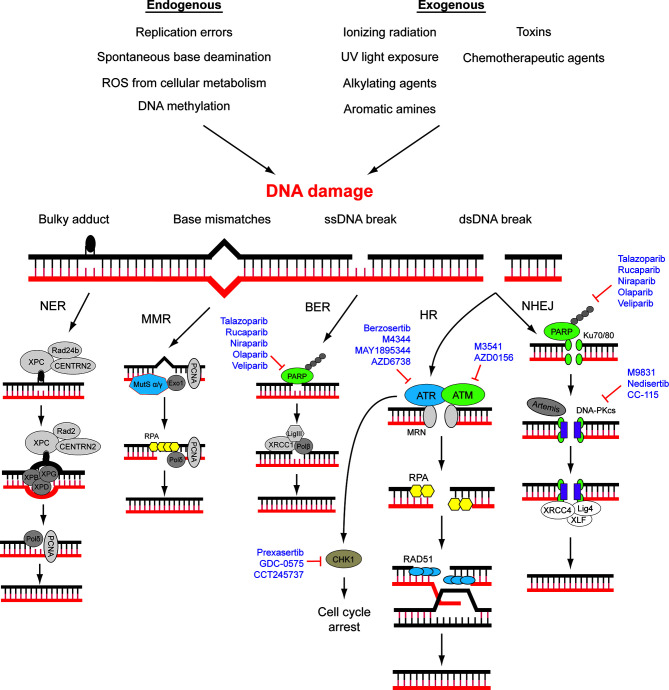
DNA damage repair (DDR) pathways and related targets for therapy. DNA damage may be caused by various environmental and endogenous stresses. Different types of DNA damage are repaired by specific signaling and repair pathways. A number of drugs that target different components of DDR pathways are approved for use or in clinical trials. NER, nucleotide excision repair; MMR, mismatch repair; BER, base excision repair; HR, homologous recombination; NHEJ, Non-homologous end-joining. Modified from (10, 11).

Components of the DNA damage response (DDR) are rich sources of molecular targets for cancer therapy (9) (**Figure 1**). DDR is a collection of interdependent signaling pathways, and machineries evolved to cope with DNA damage, which happens constantly in every cell (12). Activation of DDR can manifest into cell-cycle arrest, regulation of DNA replication, and the repair or bypass of DNA damage, ultimately alleviating the devastating effect of replicating damaged DNA. In the event of unsuccessful or suboptimal repair of DNA leading to unsustainable genomic instability, DDR can signify senescence or elimination of the affected cells by programmed cell death (13–16). DNA can be damaged in multiple ways, and thus there are multiple interrelated DDR pathways (reviewed below). Intriguingly, DDR activation is an early event during tumorigenesis (14, 15), whilst defects in one or more DDR pathways are a hallmark of cancer, leading to genomic instability and a greater dependency on the remaining pathways for survival (17). While some DDR-targeting agents, mainly of the Poly (ADP-ribose) polymerase (PARP) inhibitors (PARPi) class, have been approved for management of ovarian cancers, relatively little was known about the utility of other DDR-targeting agents in an OCCC-specific context. Given the distinct genetics features of OCCC, we argue that the disease should be vulnerable to many more DDR-targeting agents. The rationale and agents for targeting DDR have been excellently reviewed previously (10, 17–20). Here in this article, DDR and its role in cancer therapy will be briefly reviewed, then we will recapitulate the preclinical studies pointing to the potential of DDR targeting in OCCC treatment, with emphasis on the role of ARID1A in DDR.

## DNA Damage Repair Pathways and Cancer

The genomic DNA of eukaryotes is highly folded, organized, and wrapped around histones in the form of chromatin in the nucleus of mammalian cells (21). Chromatin fibers possess different degrees of compression and multiple types of chemical modifications, ensuring the accurate gene expression and the integrity of genomic structure (21, 22). DDR plays a pivotal role in the maintenance of human genomic integrity and stability through repairing and reducing DNA damage resulting from a wide variety of endogenous and exogenous threats, including ultraviolet light, ionizing radiation, topoisomerase agents, reactive oxygen species, and error-prone (non-proofreading) DNA polymerases (12, 23–26). DNA damage generates aberrant nucleotides or nucleotide fragments in the DNA strands. Five distinct DNA repair pathways have been identified in mammalian cells, including base excision repair (BER), mismatch repair (MMR), and nucleotide excision repair (NER) for single-strand break, homologous recombination (HR) and non-homologous end joining (NHEJ) for double-strand break (12, 27) (**Figure 1**). Deficiencies in DNA damage repair system may cause increased risks of generating mutations and chromosomal instability, which may ultimately lead to tumorigenesis (28). The molecular details of DDR pathways have been extensively studied, and readers are referred to a number of excellent reviews (10, 17, 27). Here, the key players of HR and NHEJ will be briefly introduced since their mechanisms and interactions are underlying determinants of cancer cell sensitivity towards some current DDR-targeting drugs, namely, PARP inhibitors.

### Non-Homologous End Joining and Homologous Recombination

DNA double-strand break (DSB) is the most severe type of DNA damages occurring within eukaryotic cells, mainly caused by the irradiation and chemotherapeutic agents (29). Irreparable DSB may lead to cell death, while its aberrant repair may result in changes of heredity materials such as chromosome deletion and translocation (30). DSB repair is generally performed through two main mechanisms: non-homologous end joining (NHEJ) and homologous recombination (HR).

In the NHEJ pathway, Ku70/80 heterodimer is responsible for recognizing and binding the ends of the DSB, acting as a scaffold to recruit other NHEJ components to the DSB for the DSB ends-processing, including DNA-dependent protein kinase catalytic subunit (DNA-PKcs), X-ray repair cross complementing 4 (XRCC4), LIG4, XRCC4-like factors (XLF), and Aprataxin and PNK-like factors (APLF) (29, 31). Once the sites of DSB are detected and secured, several NHEJ repairing proteins, including Artemis, Polynucleotide kinase/phosphatase, APLF, and Werner syndrome protein, are recruited to remove non-ligatable end groups and excise the naked strands. Then, the gap is filled in by Family X polymerase μ/λ and LIG4 (29).

The triggering of the HR pathway initiates with the formation of MRN complex (MRE11-RAD50-NBS1) for recognizing and binding to the DSB sites (32). Subsequently, several HR components, including ataxia-telangiectasia mutated kinase (ATM), CtIP, and BRCA1, are recruited to initiate a series of HR events including phosphorylation of MRE11, MDC1, and H2AX (33). The initial nucleotides excision is performed by the MRN complex through its endonuclease activity in the presence of CtlP. Then, under the promotion of BRCA1, nucleases Exonuclease 1 and DNA2 thoroughly cut the 5’ end and generate a RPA-coated single stranded DNA (ssDNA) at the 3’ end (34). Next, the BRCA1-PALB2-BRCA2 complex assembles the recombinase RAD51 onto the ssDNA to form presynaptic filaments. RAD52 also interacts with RPA and promotes RAD51 to displace the RPA wrapped on ssDNA (32, 35). Subsequently, the filament invades the homologous sequence, initiating the remaining re-synthesis of damaged DNA strands using the sister chromatid as a template for error-free repair.

NHEJ can be activated in different cell types and at various cell cycle stages but mainly in G0 and G1 phases, while HR pathway occurs mainly in the S and G2 phases (34). NHEJ pathway can quickly repair up to 85% of DNA double-strand break damage generated by irradiation. However, NHEJ is an extremely error-prone repairing mechanism since it reconnects broken DNA strands together through end processing without a DNA template and some DNA fragments might be lost during this sort of end processing (29). In contrast, HR pathway may be the most important repairing pathway as it utilizes undisturbed sister chromatid as template DNA to restore the original DNA sequence in a high-fidelity manner (35).

### Synthetic Lethality of HR-Deficient Phenotype

A cancer cell with deficiency in a DDR pathway is dependent on another DDR pathway for survival, potentiating single-agent activity of an inhibitor of that dependent pathway—an approach that has been described as synthetic lethality. Since the deficiency is specific to cancer cells, such approach is expected to spare normal cells and provide superior specificity in comparison to conventional chemotherapies (36, 37). Up to now, the most successful example of therapeutic agents realizing the synthetic lethality principle is the pharmaceutical inhibition of poly (ADP-ribose) polymerase 1 (PARP1) (37). PARP1 is a key enzyme for the detection of single-strand DNA breaks (SSBs). Inhibition of PARP1 is synthetic lethal with mutations in the genes encoding the HR proteins BRCA1, BRCA2, as well as partner and localizer of BRCA2 (PALB2). Mutations of these genes are found in small percentage of sporadic and significant proportion of hereditary high-grade serous ovarian cancer (38). A number of PARP inhibitors (PARPis) such as olaparib, rucaparib, and niraparib have been approved for the management of ovarian cancers in various settings (39–45) (**Figure 1**). Efforts are also underway to expand the experience acquired from the use of PARP1 inhibitors into a broader range of defects in HR (46).

### Predictors of Response to PARP Inhibitors

Tremendous effort has been put on extending the utility of PARPis in treating tumors without BRCA1/2 mutations, as HR deficiency may be resulted from defects in HR genes other than BRCA1/2, or through epigenetics mechanisms such as promoter methylation of BRCA1/2 (47). Thus, various approaches have been explored to identify biomarkers for PARPi sensitivity such as sequencing all known HR genes, multiplex-ligation-dependent probe amplification (MLPA) (48), promoter hypermethylation assays (47, 49), gene expression signatures (50–52), candidate single genes expression, e.g., SLFN11 (53), gross chromosomal structural changes (54–56), and direct assay for HR functioning and PARPi sensitivity such as RAD51 foci detection and dose-response test on patient-derived tumor organoids (57–60). Nowadays, identification of patients who will most likely benefit from PARPi treatment is achieved through the use of next-generation sequencing (NGS) of targeted gene panels or genomic signatures (genomic scars). International consensus is still lacking regarding the optimal approach to define HR deficiency (60).

Currently, there are five commercially available FDA-approved CDx tests for evaluating PARPi eligibility: BRACAnalysis CDx and MyChoice CDx from Myriad Genetic Laboratories, Inc., as well as FoundationOne CDx, FoundationFocus CDxBRCA, and FoundationOne Liquid CDx from Foundation Medicine (60, 61). NGS-based CDx tests follow similar workflows: DNA is extracted from tissues and sequenced through NGS platforms, and then raw reads are processed through proprietary bioinformatics pipelines for variants calling and medical interpretation. For instance, according to the ACGM classification, variants can be classified as: pathogenic (class 5), likely pathogenic (class 4), variant of uncertain significance (class 3), likely benign (class 2), and benign (class 1) (62). The five CDx mentioned can all detect pathogenic or likely pathogenic variants of BRCA1/2. Among them, MyChoice CDx and FoundationOne CDx assays are prospectively validated assays for evaluation of HR deficiency status (61). The HR deficiency status can be assessed by checking the percentage of genomic regions with loss of heterozygosity (LOH) using single nucleotide polymorphism (SNP) sequencing or by calculation of the Genomic Instability Score (GIS) *via* coalescing three parameters: LOH, large scale transitions (LSTs) and telomeric allelic imbalance (TAI). Other assays such as the FDA-approved MSK-IMPACT, which provides integrated mutation profiling of actionable cancer targets developed by Memorial Sloan Kettering’s Department of Pathology, also cover detection of somatic variants in DDR genes and allow evaluation of experimental HR deficiency scores (63, 64). Recently, HRDetect, a new computational method dedicated to measuring HR deficiency based on whole-genome sequencing (WGS) data, is gaining momentum as a predictor of PARPi response. The HRDetect algorithm extracts a tumor’s mutational signatures and identify those specific for HR deficiency, such as multiple small duplications throughout the genome or numerous large tandem duplications (65). HRDetect successfully identified HR-deficient triple negative breast cancer (TNBC) and predicted their rucaparib sensitivities in a translational clinical trial RIO (EudraCT 2014-003319-12) (66). The algorithm is easily accessible through the reference web-based tool Signal (67).

The benefits of these CDx tests and related technical platforms were supported by clinical trials. In the SOLO1 trial (NCT01844986), patients with newly diagnosed advanced ovarian cancers were tested for BRCA1/2 mutation by BRACAnalysis (Myriad) or BRCA1/2 genetic testing assay (BGI) before randomization into olaparib and placebo groups as maintenance therapy. The risk of disease progression or death was 70% lower with olaparib than with placebo (68). NGS platform from Foundation Medicine was adopted to evaluate BRCA mutation status and LOH in patients with platinum sensitive recurrent ovarian high-grade serous carcinomas (44). The findings that patients with BRCA mutant or BRCA wild-type and LOH high cancers treated with rucaparib showed longer progression-free survival than patients with BRCA wild-type LOH low cancers support the efficacy of evaluating LOH in cancers to identify BRCA wild-type patients who are likely to benefit from rucaparib therapy. LIGHT (NCT02983799) is a phase 2 multicenter study to assess the efficacy and safety of olaparib in patients with BRCA mutation and HR deficiency status evaluated by BRACAnalysis CDx and myChoice CDx tests. Four cohorts were identified: subjects with germline BRCA mutations (gBRCAm), somatic BRCA mutations (sBRCAm), or potential aberrations in homologous recombination deficiency (HRD) (HRD-positive), as well as in subjects without identifiable HRD (HRD-negative). Consistent with the maintenance therapy setting, similar efficacy of olaparib was observed among gBRCAm and sBRCAm patients. More importantly, for non-BRCAm patients, a longer median progress-free survival and higher objective response rate were observed in the HRD positive cohort compared with the HRD-negative cohort (69). Recently, short-term patient-derived ovarian cancer organoids have been proposed to serve as testing platform of real-time HR deficiency and PARPi sensitivity, capable of supplementing sequencing-based approaches (70). Most of these investigations were performed in high-grade serous ovarian cancer. There is a relative lack of studies regarding biomarkers and clinical trials involving OCCC in the literature. It will be worthwhile to investigate the general HR deficiency status of OCCC in relation to PARPi and other DRR-targeting therapies’ sensitivity.

## Clear Cell Carcinoma of the Ovary

### Epidemiology, Morphology, Immunophenotype, and Genetic Features of OCCC

The incidence of OCCC shows wide variation among different geographic populations. OCCC contributes to less than 12% in Western countries where HGSC is the most common ovarian cancer subtype. In contrast, up to 20–27% of ovarian cancers in Asian countries are OCCC (5, 71, 72). OCCC generally shows morphologic and molecular biological characteristics distinct from other subtypes of epithelial ovarian cancers. Differences in pathogenesis and genetic profiles between OCCC and other major histological subtypes of ovarian cancers are summarized in **Table 1**.

**Table 1:** Distinct features of OCC as compared with other major histotypes of ovarian cancer.

	Clear cell carcinoma	Endometrioid carcinoma	High-grade serous carcinoma	Low-grade serous carcinoma	Mucinous carcinoma
**Precursor lesions (2)**	Endometriosis	Endometriosis	Serous tubal intraepithelial carcinoma (STIC)	Serous borderline tumor	Mucinous borderline tumor
**Genetic predisposition (73, 74)**	Lynch syndrome	Lynch syndrome	BRCA1/2 mutations	–	–
***TP53* status (38, 75)**	*TP53* wild-type	*TP53* mutations uncommon	*TP53* mutant almost ubiquitous	*TP53* wild-type	Both *TP53* mutant and wild types exist
**Frequent mutations and genetic changes (76–80)**	*ARID1A*, *PIK3CA*, *PTEN*, microsatellite instability	*ARID1A*, *PIK3CA*, *CTNNB1*, *PTEN*, microsatellite instability	Genomic instability	*KRAS*, *NRAS*, *BRAF*, *ERBB2*	*KRAS*, *ERBB2* amplification

Modified from (81).

**Figure 2:**
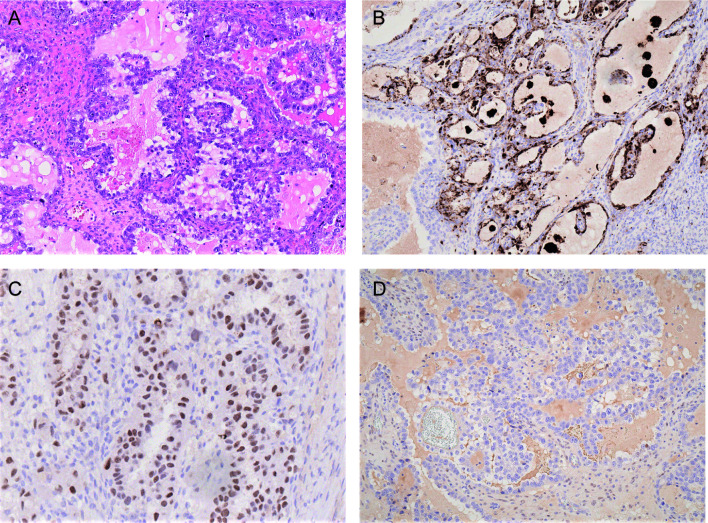
Photomicrographs of OCCC showing **(A)** tubulocystic pattern with tubules lined by hobnail, clear and eosinophilic cells (H&E). The carcinoma cells are immunoreactive for napsin A **(B)** and HNF-1β **(C)** but negative for estrogen receptor **(D)**.

Morphologically, OCCCs are mainly composed of tumor cells with clear cytoplasm forming tubule-cystic, papillary, and solid architectures. Tumor cells with eosinophilic cytoplasm are often present. Hobnail cells may be found lining the tubules and cysts (82) (**Figure 2A**). OCCC usually shows strong positive immunoreactivity for cytokeratin 7, PAX8, HNF-1β, and Napsin A but are often negative for estrogen receptor (ER), progesterone receptor (PR), and Wilms tumor protein 1 (WT1) (83–86) (**Figures 2B–D**). Assessment of the morphology and immunoprofile is important to differentiate OCCC from other histological subtypes of ovarian carcinomas, particularly high-grade serous carcinoma and endometrioid carcinoma.

Genetically, loss of heterozygosity at chromosome 3p25 region is observed in nearly half of primary and metastatic OCCC (87). The high incidence of distinct genetic alterations, including mutations in *ARID1A* (46–66.7%) (76, 77, 88) and *PIK3CA* (32–50%) (88–90), as well as loss of PTEN activity (28–38%) (91, 92), are recognized to be key driving events in the pathogenesis of OCCC. In comparison with high-grade serous carcinoma, OCCC seldom exhibits mutations in *TP53* and *BRAF* genes (90, 93). In addition, upregulated expression of hepatocyte nuclear factor-1β (HNF-1β) is commonly reported in ovarian clear cell tumors, including borderline tumors and carcinomas, suggesting a critical role of HNF-1β in the tumorigenesis of OCCC (72, 94, 95). OCCC also shows a high expression level of vascular endothelial growth factor (VEGF) that is commonly associated with poor clinical outcome (96). Compared to other subtypes, OCCC is more often diagnosed at an early stage, and the prognosis is good when no metastasis occurs (97). However, OCCCs in advanced stages or recurrent status usually show poor prognosis and are commonly resistant to most of existing chemotherapeutic treatments (6). Hence, exploring novel predictive biomarker and efficient therapeutic strategy is badly needed for improving the survival of OCCC patients.

### DDR Status in OCCC

A significant proportion of OCCC shows HR deficiency and hence should be susceptible to PARP inhibitor therapy. Using targeted capture and deep sequencing analysis for germline and somatic loss-of-function mutations in 30 genes, including *BRCA1*, *BRCA2*, and 11 other genes in the HR pathway in 390 ovarian carcinomas, Pennington et al. documented that 26% of OCCC in their cohort harbored HR-related mutations (98). Similarly, about one-third of a Japanese cohort of OCCC was reported to have germline and somatic mutations in HR genes. The percentage is lower than that of high-grade serous carcinoma in which 44% present such mutations. Notably, the lists of mutated HR genes in high-grade serous carcinoma and CCC exhibited significant differences, with *ATM* being the most frequently mutated HR gene in OCCC (99). Moreover, as much as half of the OCCC exhibited the BRCAness phenotype (mutation in 18 different HR genes or having *BRCA1* hypermethylation or loss of BRCA1 protein expression) in a Danish ovarian cancer cohort comprising 20 cases of OCCC (100). Genetic abnormalities of OCCC have therefore been suggested to be synthetic lethal with PARP inhibitors (5).

Some OCCCs are suggested to have defective DNA mismatch repair (MMR), and Lynch syndrome confers susceptibility to OCCC (101, 102). Indeed, endometrioid and clear-cell carcinomas are the most common tumors in the ovary to be associated with Lynch syndrome (103–105). By assessing microsatellite markers, Cai et al. concluded in an earlier study that about 21% of their OCCC samples exhibited at least some levels of microsatellite instability (MSI). A strong correlation existed between alterations in the expression of *hMLH1* and *hMSH2*, two major DNA MMR genes, and the high level of MSI (MSI-H) in these tumors (106). Bennett et al. evaluated the expression MMR proteins in 109 unselected OCCC samples with known clinical characteristics. In their cohort, 6 (5.5%) of the tumors exhibited loss of MMR proteins (107). Howitt et al. reported that MSI-H or loss of MMR protein expression can be detected in 10% of OCCC and that these tumors with MSI-H status are associated with increased tumor-infiltrating lymphocytes and increased PD-L1 expression, thus should be susceptible to immune checkpoint therapy (108). However, PD-L1 expression was found to be more prevalent among OCCCs when compared with endometrial CCC irrespective of their MSI status (109). In another study, exome sequencing of 48 pairs of OCCC tumor/non-tumor samples identified 3 (6.25%) cases showing somatic hypermutated phenotype with ≥12-fold somatic mutations than the other 45 cases. These hypermutated OCCC cases have 8.1-fold higher frameshift indels/non-frameshift indels ratio indicating faulty MMR mechanisms consistent with MSI, leading to elevated single nucleotide indel insertion or deletion (88).

Compared with HR and MMR, less is known about the NER or NHEJ capability of OCCC. The mRNA levels of endonuclease ERCC1 and helicase XPB (ERCC3), key players in the NER pathway, tend to be higher in OCCC as compared to other types of ovarian cancers (110). Elevated ERCC1 is also suggested to contribute to chemoresistance in ovarian cancer (108), although the number of OCCC in the study was very small.

### *ARID1A* Is a DDR Component and Promising Target of Synthetic Lethality Therapy

**Figure 3:**
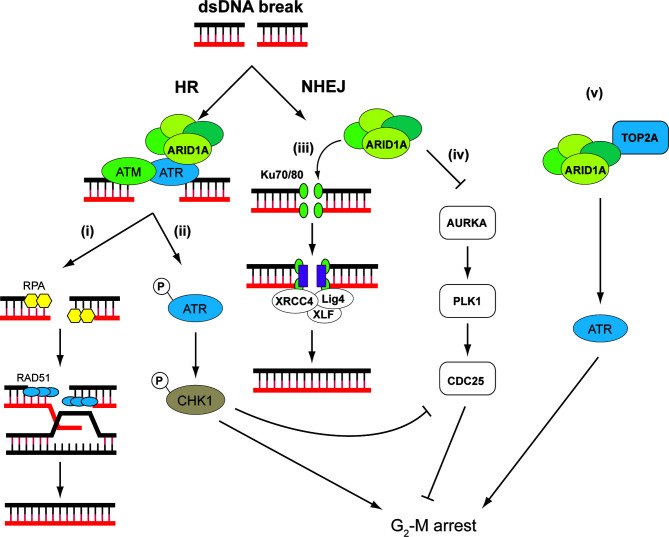
ARID1A is involved in DDR through different mechanisms. ARID1A is implicated on both HR and NHEJ. On DNA double-strand breaks (DSB), ATR directly interacts and recruits ARID1A containing SWI/SNF complex, which serves to (i) reduce nucleosome density at the DSB and facilitate DNA end resection to create single-strand for RPA deposition. The RPA-covered single-strand DNA is necessary for RAD51-mediated homology search and strand invasion (116). (ii) In addition, ARID1A is necessary for ATR activation, which, through CHK1, signals G_2_-M arrest (117). (iii) In NHEJ, SWI/SNF complex is necessary for recruitment of Ku70/80 and XRCC4 at DSB. Thus, loss of ARID1A leads to reduced NHEJ activity (118). (iv) ARID1A transcriptionally represses the mitosis driver kinase AURKA. In cells with ARID1A loss, AURKA-PLK1-CDC25C pathway is upregulated. In addition, CDC25C activity is negatively regulated by ARID1A–ATR–CHK pathway under DNA damage conditions, thereby strictly controlling the CDC25 activity in ARID1A WT cells. CDC25C activity is hence de-repressed in ARID1A mutated cells, leading to weakened G2-M checkpoint (119). (v) In addition, SWI/SNF complex may recruit TOP2A to chromatin, which, through ATR and other mechanisms, may signal S and G2-M arrest. These factors might render tumor cells sensitive to small-molecule ATR inhibitors as these agents impair the ability of cells to mount adequate DDRs while at the same time accelerating mitotic entry (120).

*ARID1A* is a subunit of the SWI/SNF chromatin-remodeling complex encompassing multiple proteins including also ARID1B, SMARCA4, and SMARCB1 that have tumor-suppressor functions (111). By altering chromatin structure and presumably modulating the accessibility of DNA, SWI/SNF complexes assume important roles in controlling DNA replication, transcription, and repair (112). Consistent with this view, SWI/SNF complexes or some of their components were found to be recruited to damaged DNA early in the repair process (113–115). Of note, *ARID1A*, which is frequently mutated in OCCC (38), and its homolog *ARID1B* participate in cellular resistance to various types of DNA damage, including DSB (**Figure 3**).

ARID1A and ARID1B are two mutually exclusive subunits in the SWI/SNF complex. Knocking down ARID1B in ARID1A-deficient colorectal cancer cells sensitized the cells to DNA damage caused by irradiation (121). It is therefore proposed that ARID1B depletion can work with radiotherapy to enhance response of patients with ARID1A-deficient malignancies. ARID1A and ARID1B accumulate at DNA damage site, and suppression of their expression reduces NHEJ activity. Several other components of the SWI/SNF complex including SNF5, BAF60a, BAF60c, BAF155, and BAF170 exhibit a similar NHEJ attenuated phenotype (118).

*ARID1A* mutation is prevalent in cancers, and synthetic lethal therapies associated with these mutations are being actively explored (122). For instance, it has been reported that ARID1A-deficient HEC151 uterine endometrial cancer and TOV21G OCCC cell lines acquire high sensitivity to PARP inhibition after exposure to exogenously induced DNA breaks such as ionizing radiation (123). A higher level of DSBs and cell death, demonstrated by immunoreactivity for phosphor-H2AX and cleaved caspase-3 respectively, was found in ARID1A-deficient cancers after combined radiation and PARP inhibitor therapy.

Moreover, in endometrial atypical hyperplasia, which demonstrates heterogeneous and patchy loss of ARID1A, higher immunoreactivity for phospho-H2AX, a marker for DSBs, was found in ARID1A-negative tumor foci than in areas with intact ARID1A expression. Immortalized endometrial epithelial cells with ARID1A knockdown also had lowered NHEJ activity and consequently higher susceptibility towards irradiation and PARP inhibitor olaparib (123). Indeed, the ARID1A-deficient CCC cell line TOV21G exhibited sensitivity towards olaparib, whereas the ARID1A wild-type CCC line RMG1 was apparently refractory to the drug (123). NUCOLL43, a novel OCCC cell line derived from an ARID1A-positive tumor, also exhibited intact DNA HR capacity (124).

ATR, a serine/threonine-specific protein kinase also known as ataxia telangiectasia and Rad3-related protein, is pivotal in detecting DNA damage and activating DNA damage checkpoint response (125, 126). Oncogene activation can impose replication stress on tumor cells, which may rely on ATR checkpoint function for survival. This suggested a rationale for applying ATR inhibitors as anticancer drugs (127). Potent and specific ATR inhibitors have been discovered including EPT-46464, AZ20, VE-821, and VX-970, some of which have entered clinical trials (120).

In the colorectal carcinoma cell line HCT 116, ARID1A is recruited to DSBs by its interaction with ATR where it sustains ATR activation and facilitates the generation of RPA coated single-strand DNA. Loss of ARID1A leads to impaired checkpoint activation and repair of DNA DSBs, which sensitizes cells to DSB-inducing agents such as radiation and PARP inhibitors (117).

Screening of genetic profile synthetic lethal with the ATR inhibitor VE-821 also identified ARID1A suppression as contributing factor to cytotoxicity of VE-821. ARID1A deficiency results in topoisomerase 2A and cell cycle defects, which cause an increased reliance on ATR checkpoint activity. Such mechanistic impact sensitizes ARID1A deficient triple-negative breast cancer cells to clinical inhibitors of ATR, both *in vitro* and *in vivo (*120). Indeed, a recent phase I clinical trial of an ATR inhibitor VX-970 on various advanced solid malignancies including cancers of the ovary, breast, colon, and stomach as monotherapy or in combination with carboplatin showed that the drug was well tolerated and showed satisfactory response (128). Mechanistically, significant inhibition of phosphorylation of CHK1, an ATR downstream substrate, was demonstrated.

Similarly, in germ cell tumors, ARID1A deficiency produced by CRISPR/Cas9 gene editing or pharmacological inhibition considerably sensitized tumor cells towards ATR inhibition (129). Mechanistically, ARID1A was postulated to control gene transcription, DDR, and epigenetic profile through putative downstream effectors DNA methyltransferase 1-associated protein DMAP1 and DNA Polymerase POLE. These data suggested ATR inhibition to be a promising approach to work with ARID1A mutations and warrant further investigation in OCCC.

Aurora kinases are serine/threonine kinases controlling multiple functions in mitosis including chromatid segregation. These kinases are related to the G1 DNA damage checkpoint *via* the p53 and p73 pathways. Aurora kinase A (AURKA) or serine/threonine protein kinase 6, triggers G2/M transition by phosphorylating a number of substrates including Polo-like kinase-1 (PLK1), ultimately leading to the nuclear localization of cell division cycle 25C (CDC25C). AURKA overexpression is common in many malignancies including leukemia as well as cancers of the ovary, lung, pancreas, liver, and colon (119). Hence, AURKA inhibitors are actively being investigated as cancer therapeutics (130). Inhibition of AURKA may contribute to the G2 DNA damage checkpoint through AURKA’s effect on PLK1 and CDC25B activation (131). Intriguingly, we have earlier reported high expression of PLK1 in OCCC, and PLK1 was shown to regulate apoptosis and autophagy of OCCC cells in relation to chemosensitivity (132).

ARID1A can attenuate *AURKA* transcription *via* epigenetic mechanism. On the other hand, ARID1A has a synthetic lethal interaction with AURKA in colorectal cancer cells such that *AURKA* inhibition can selectively impede the growth of ARID1A-deficient colorectal cancer cells. HCT116 with ARID1A knockout was most sensitive to AURAKA inhibitors in a screen of epigenetic drug library. Interestingly, knockout cells were more sensitive to AURKA inhibitors than PARP inhibitors. The ARID1A knockout genetic background aggravated the abnormal chromosome arrangement and segregation of AURKA inhibition and subsequently more apoptosis (119). In cells lacking ARID1A, AURKA upregulation leads to persistent activation of CDC25C (119). Hyperactivation of AURKA/PLK1/CDC25 axis was therefore observed in ARID1A knockout cells as ARID1A was found to be a transcription repressor of AURKA (119).

OCCC is the malignancy with highest proportion of *ARID1A* mutation (~50%). Given the fact that ARID1A interacts closely with key players in several DDR pathways, we hypothesize that most OCCC will be responsive to DDR-targeting drugs synthetic lethal to ARID1A mutations.

### PI3K/AKT Pathway Alterations in OCCC May Enhance DDR Therapy

Compared with other histologic subtypes, OCCC exhibits higher prevalence of *PIK3CA* mutations and *PTEN* deletion (20–46% and 20%, respectively, compared with 2.3–3.7% and 7%, respectively, in high-grade serous ovarian carcinoma) (133). However, using PI3K pathway inhibitors as monotherapies for ovarian cancer was only met with limited success (8). Hence a new paradigm for utilizing PI3K inhibitors in ovarian cancer management is to explore combination strategies to improve the efficacy of PI3K pathway blockades (134). Since the PI3K/Akt/mTOR signaling pathway exerts multiple layers of regulation on the repair of DNA DSBs and SSBs, it has been suggested that DDR proteins may represent attractive targets of synthetic lethality with PI3K inhibitors (134). Indeed, it has been shown that PI3K inhibitors and CHK1 inhibitors combination treatments exhibit remarkably higher cytotoxicity in high-grade serous ovarian carcinoma cells compared with each individual drug alone, with evidence of increased DNA damage, chromosomal breaks, and mitotic catastrophe (135).

While targeting of PI3K/Akt/mTOR in *in vitro* models of OCCC has shown promising effectiveness (136–140), little is known about the impact of the targeting agents on the DDR capacity of OCCC cells. Since hyperactivation of *PI3K3A* and *AKT via* gain-of-function mutations is common in human cancers, perhaps insight could be drawn from investigations in other malignancies. In cervical cancer, PIK3CA mutation and increased expression of pAKT have been found to be related with resistance to radiotherapy (141, 142). Activated AKT can stimulate DNA repair such as DSB repair after radiotherapy (143). Common hotspot mutation of PIK3CA, PIK3CA-E545K mutation has also been found to enhance DNA repair in cervical cancer cells as demonstrated by fewer pH2A.X foci and more highly activated Chk1/Chk2, regulators of DDR in mutated cells (144). On the other hand, PI3K inhibitor (LY294002) can, through DSB repair, sensitize cervical cancer cells to radiation *in vivo* and *in vitro (*145–147).

The PI3K/AKT and Wnt/β-catenin pathways interact in the impact on DDR. PIK3CA-E545K is reported to confer resistance to ionizing radiation in cervical cancer cells by inducing overexpression and nuclear accumulation of β-catenin (144). Inhibiting β-catenin enhances radiosensitivity of cervical cancer cells *in vitro* and *in vivo* particularly in PIK3CA-E545K mutated cells. The Wnt/β-catenin inhibitor XAV-939 boosted radiation-induced DNA damage but attenuated DDR (144).

The activity of these pathways may also be related to the MMR status of the malignancies. The MMR-deficient colorectal carcinoma was found to be more related to PI3K, DDR, and WNT pathway aberrations, a profile different from MMR proficient cancer cells. Among pivotal genes in the WNT pathway, mutual exclusivity between mutations of *CTNNB1* and APC or RNF43 was also demonstrated. It is interesting since *CTNNB1* gene encoded β-catenin, the major effector of the WNT signal pathway, is negatively controlled by APC and RNF43. Moreover, MLH1-methylated MMR-deficient carcinomas have less *CTNNB1* mutations than MLH1-unmethylated MMR-deficient cancers (148).

The PI3K/AKT and Wnt/β-catenin pathways also play a role in synergistic lethality in cancer therapy. In gastric cancer, synergistic lethality for combination therapy with paclitaxel and PI3K p110α-specific inhibitor alpelisib was found to be stronger in PIK3CA-mutant cells related to enhanced DDR and apoptosis, as demonstrated by γ-H2ax and caspase 3/7 assays, respectively (149). Indeed, Juvekar et al. have demonstrated that PI3K3CA inhibition of breast cancer cells by alpelisib can produce raised nucleotide depletion-mediated DNA damage and thus death of cancer cells (150). PI3K3CA inhibition was also found to be more effective in inducing DNA damage than inhibition of AKT.

Since aberration of these pathways are common in OCCC, exploration of DDR-targeted therapy in relation to the PI3K/AKT and Wnt/β-catenin pathways in OCCC may trigger novel treatment approaches

### HNF-1β and DDR

Overexpression of HNF-1β is a clinically useful marker for discriminating OCCC from other subtypes of ovarian cancer (151). As a transcription factor HNF-1β assume several functional roles during the carcinogenesis of OCCC, such as metabolic reprogramming of the cancer cells (152, 153), causing tumor-associated thrombosis (154), and conferring carboplatin resistance (155). More importantly, HNF1β appears to be integral to the transformation of endometriosis lesions, serving as the master regulator of an antioxidant detoxification system responsible for resisting the oxidative microenvironment caused by hemolysis during the development of endometriosis (156). In terms of DDR, HNF-1β takes part in the Chk1 regulated cell cycle checkpoint in endometriotic cells, contributing to their transformation (157). Similarly, HNF-1β activates Chk1-mediated cell checkpoint in the presence of DNA damaging agents such as bleomycin and thereby enhancing cell survival by maximizing DNA repair efficiency (158). CHK1 inhibitor can abolish such activity and hence sensitize cells to the chemotherapy (159). These studies suggested HNF-1β is involved in the DDR of OCCC and that DDR targeting may be effect in HNF-1β overexpressing tumors.

## Further Evidence of DDR-Targeting Drugs’ Efficacies in OCCC

The utilities of DDR-targeting drugs as cytotoxic agents of OCCC cells have been evaluated in several preclinical models. By assessing RAD51 foci formation in the presence of ionizing radiation-induced DNA DSBs, Wilkerson et al. categorized a panel of 12 OCCC cell lines as HR-competent and HR-deficient. The HR-deficient cells were also demonstrated to exhibit increased susceptibility towards cisplatin and the PARP inhibitor BMN-673 when compared with the HR competent cancer cells (160). Moreover, loss of PTEN function was found to be related to HR DNA repair deficiency in a portion of OCCC cells. Indeed, it is known that PTEN plays a crucial role in DDR in several cancers such as colorectal cancers, and PTEN polymorphism was associated with response to neoadjuvant chemoradiotherapy (161).

## Predictors of Response to PARP Inhibitors and Immunotherapy for OCCC

According to the recent guideline from the American Society of Clinical Oncology (ASCO) (162), all women with ovarian cancers irrespective of histological subtypes should have germline genetic tests of ovarian cancer susceptibility genes as a multigene panel that should at least include *BRCA1*, *BRCA2*, *RAD51C*, *RAD51D*, *BRIP1*, *MLH1*, *MSH2*, *MSH6*, *PMS2*, and *PALB2*. Somatic genetic testing on tumor tissue should be performed if germline pathogenic or likely pathogenic variants cannot be found. It is considered that such genetic testing should not be restricted to high-grade serous carcinoma (98, 163) since women having clear cell, endometrioid, low-grade serous, or carcinosarcoma subtypes of ovarian cancer were also found to still carry a significant risk of harboring germline BRCA mutation near to that of high-grade serous carcinoma (98).

MMR deficiency is found in about 10–12% of unselected ovarian cancers although it is more common in non-serous histological subtypes (101, 104, 164). It is reported that 11.5% of clear cell carcinoma exhibit MMR deficiency. Microsatellite instability is also demonstrated in a subset of OCCC, rendering them immunogenic (108). Hence, routine testing of MMR deficiency in OCCC is also recommended for consideration of treatment with pembrolizumab in the setting of recurrent disease.

## Conclusion

OCCC is characterized by chemoresistance and worse prognosis particularly cases with extraovarian dissemination demanding novel therapeutic approaches, especially targeted therapy. Like other solid tumors, OCCC exhibits frequent DDR defects that can be exploited to specifically kill cancer cells, under the principle of synthetic lethality. In fact, one may expect that DDR targeting can be exceptionally effective given the distinct genetic characteristics of the malignancy. While PARP inhibitors have proven utilities in high-grade serous ovarian carcinomas, the efficacies of other DDR-targeting approaches in OCCC await exploration, particularly by comprehensive clinical trials. Biomarkers for predicting sensitivity to PARP inhibitors and other DDR-targeting strategies as well as identifying synergistic combinations may readily be applicable in OCCC and worth exploring.

## Author Contributions

OW and AC conceived and wrote the manuscript. JL wrote the manuscript. All authors contributed to the article and approved the submitted version.

## Conflict of Interest

The authors declare that the research was conducted in the absence of any commercial or financial relationships that could be construed as a potential conflict of interest.

## Publisher’s Note

All claims expressed in this article are solely those of the authors and do not necessarily represent those of their affiliated organizations, or those of the publisher, the editors and the reviewers. Any product that may be evaluated in this article, or claim that may be made by its manufacturer, is not guaranteed or endorsed by the publisher.
